# Associations Between Abstract Concepts: Investigating the Relationship Between Deictic Time and Valence

**DOI:** 10.3389/fpsyg.2021.612720

**Published:** 2021-02-12

**Authors:** Barbara Kaup, Nina Scherer, Rolf Ulrich

**Affiliations:** Department of Psychology, University of Tübingen, Tübingen, Germany

**Keywords:** abstract concepts, metaphoric mapping, time, valence, spatial representation, sentence continuation, compatibility

## Abstract

The present study examines whether deictic time and valence are mentally associated, with a link between future and positive valence and a link between past and negative valence. We employed a novel paradigm, the two-choice-sentence-completion paradigm, to address this issue. Participants were presented with an initial sentence fragment that referred to an event that was either located in time (future or past) or of different valence (positive or negative). Participants chose between two completion phrases. When the given dimension in the initial fragment was time, the two completion phrase alternatives differed in valence (positive vs. negative). However, when the given dimension in the initial fragment was valence, the two completion phrase alternatives differed in time (future vs. past). As expected, participants chose completion phrases consistent with the proposed association between time and valence. Additional analyses involving individual differences concerning optimism/pessimism revealed that this association is particularly pronounced for people with an optimistic attitude.

## Introduction

According to embodied cognition accounts, cognitive processes operate on meaning representations that are grounded in experience and are therefore sensorimotor in nature (e.g., Zwaan and Madden, 2005; Barsalou, 2008). One of the central issues plaguing these accounts are abstract concepts, such as justice or imaginary numbers. Abstract concepts cannot be experienced directly and thus the question arises how abstract concepts might be grounded in experience. Advocates of so-called metaphoric-mapping accounts have suggested that abstract concepts are grounded in experience by being mapped onto concrete domains for which direct experiences exist. One such concrete domain that has been in the focus of research on metaphoric-mapping accounts is the spatial domain (e.g., Lakoff and Johnson, 1980, 2008).

As reviewed in Núñez and Cooperrider (2013) and consistent with such a metaphoric-mapping account, a bulk of studies has demonstrated that Western cultures conceptualize deictic time (which refers to past, present, and future) along the sagittal axis (front-back) and also along the lateral axis (left-right). On the sagittal axis, the past is behind and the future in front of a person (e.g., Clark, 1973a; Alverson, 1994). On the lateral axis, the past is on the left and the future on the right (e.g., Torralbo et al., 2006). Similarly, for valence, such spatial associations are assumed both for the vertical axis (up-down; Meier and Robinson, 2004) and for the horizontal axis (left-right; Casasanto, 2009). Some of these mappings manifest themselves in linguistic expressions. For instance, the sentence “*Exciting events lie ahead of us*” indicates a mapping of the future to frontal space, and “*He is feeling down today*” a mapping of negative valence to lower vertical space.

The results of several reaction time studies are consistent with the mappings of time and space mentioned above and valence and space (cf. von Sobbe et al., 2019). For instance, people are faster to distinguish between sentences describing past or future events when asked to respond to future sentences by moving a slider to the front and past sentences by moving a slider toward the back compared to the reversed S-R-mapping (Ulrich et al., 2012). Likewise, people are faster to decide between past- and future-related words if they are asked to respond with the left to the past and the right to the future compared to a reversed S-R-mapping (e.g., Santiago et al., 2007; Ulrich and Maienborn, 2010). These RT results indicate that the future is associated with the front and the right, whereas the past is associated with the back and the left. Similar results hold for valence. People are faster to judge a word’s valence when they are asked to respond to positive words by pressing an upper key and to negative words by pressing a lower key compared to the reversed S-R mapping (Dudschig et al., 2015). Likewise, right-handed people are faster to judge a word’s valence when the decision involves a left-hand response for negative valence and a right-hand response for positive valence compared to the opposite S-R mapping (de la Vega et al., 2012). These results indicate that positive valence is associated with upper vertical space as well as with the right. In contrast, negative valence is associated with lower vertical space as well as with the left, at least for right-handers.

Interestingly, as the previous paragraphs show, there seems to be a commonality between valence and time concerning the existing metaphoric mappings onto space; for both of these abstract concepts exists a mapping onto the lateral spatial axis. This commonality naturally provokes the hypothesis that there might be an association between time and valence due to their common mapping onto lateral space. If positive and future-related entities both map onto the right, and negative and past-related entities both map onto the left, then this might lead to a link between future and positive on the one hand and between past and negative on the other hand.

There is indeed ample evidence from rating studies that fit well with this hypothesis. Imagined future events are more emotionally positive and idyllic than past counterparts (e.g., Newby-Cark and Ross, 2003; D’Argembeau and Van der Linden, 2004; Berntsen and Bohn, 2010; Rasmussen and Berntsen, 2013). For example, Newby-Cark and Ross (2003) asked their participants to recall and forecast personally essential events. The anticipated events were almost always rated more positively than the remembered events (for a similar result, cf. Rasmussen and Berntsen, 2013). In a subsequent experiment, positive future events were more rapidly generated than negative future events, although past events were recalled equally rapidly independent of their valence. This suggests that future negative events are more difficult to imagine than future positive events, suggesting that future time and positive valence are associated. A study that seemingly challenges the generality of these findings was conducted by Rubin (2014). The author reported that participants rated future negative events as more troubling than past negative events. However, in this study, the context of recall was narrowed to specific categories of events (e.g., phone calls), and the author’s focus was on negative events. It seems conceivable that a focus on traumatic events let the future appear more troubling than the past. Under less restricted situations, people seem to tend to view the future as more positive than the past, and this is well in line with the hypothesis that there is an association of future time and positive valence and of past time and negative valence.

In this paper we suggest a new approach for studying the potential association between time and valence in a more implicit task that does not involve temporal or valence ratings. We presented participants with an initial sentence fragment (e.g., “*Next week I will visit*…*”)* and asked them to complete the sentence by choosing one of two possible completion phrases (e.g., “*my daughter*” or “*my dentist*,” see Table 1). The initial sentence fragments either gave partial information about a past or a future event (dimension of initial phrase: time) or contained a positive or negative content word (given dimension: valence). Specifically, when the dimension of the initial sentence was time, the dimension of the completion phrase was valence and participants were to select either a positive or a negative content word (e.g., *“Next week I will visit*… *my daughter”* vs. *“Next week I will visit*… *my dentist”).* By contrast, when the initial phrase referred to valence, the dimension of the completion phrase was time and participants were asked to choose between the past and the future (e.g., *“The tour through the bunker*… *was last week”* vs. *“The tour through the bunker*… *is next week”)*. Thus, there are four possible conditions: (a) Future: choose between positive and negative valence, (b) Past: choose between positive and negative valence, (c) Positive valence: choose between past time and future time, and (d) Negative valence: choose between past time and future time. In the following, the initial phrase dimension will be called the “given dimension.”

**TABLE 1:** Examples of the experimental sentence pairs with given dimension time and valence.

**Given Dim.**	**Given value**	**Ass. side**	**Initial fragment**	**Completion pos/future**	**Completion neg/past**
Time	Future	Right	*Next week I will visit*…	*my daughter*	*my dentist*
Time	Past	Left	*Last week I visited*…		
Valence	Positive	Right	*The tour through the palace*…	*will take place next week*	*took place last week*
Valence	Negative	Left	*The tour through the bunker*…		

*Dim., Dimension; Ass. Side, Associated Side. The latter term refers to the mapping of time and valence to space, with future and positive being mapped onto the right side and past and negative onto the left side.*

We were interested in whether participants’ choices of the completion phrase would depend on the value of the given dimension. If time and valence are indeed associated based on being part of a common metaphorical mapping onto the lateral spatial axis, then it is expected that participants choose the completion phrase that is consistent with this common mapping. More specifically, for the given dimension time, the given value future is presumably mentally mapped onto the right side (associated side = right), and accordingly, participants are expected to select the completion that is also mentally mapped onto the right side, namely the positive completion. For the given value past, which is mentally mapped onto the left side (associated side = left), participants can be expected to choose the completion that is also mentally mapped onto the left side, namely the negative completion. Similarly, for the given dimension valence, the given value positive is presumably mentally mapped onto the right side (associated side = right), and accordingly, participants should tend to choose the completion that is also mentally mapped onto the right side, namely the future completion. For the given value negative, which is presumably mentally mapped onto the left side (associated side = left), participants can be expected to choose the past completion.

We also speculated whether the presumed association between time and valence would be stronger for people with an optimistic personality than for people with a pessimistic personality. We reasoned that his might be the case because optimists are defined to be people “who are inclined to be hopeful and to expect good outcomes” (Merriam Webster)^1^. In the terminology of the present manuscript, optimists can be conceptualized as people who assign positive valence to future events. To gain more information with respect to this possibility, we included a scale measuring optimism and pessimism (SOP2) at the end of the experiment and included the measure as a covariate in a *post hoc* analysis of the results.

Thus, to summarize: If time and valence are associated, with future time being linked to positive valence and past time being liked to negative valence, then we would expect to observe a main effect of the factor Associated Side. Right-side choices should be more frequent when the initial sentence fragments are associated to the right side (positive valence or future time) compared to when they are associated to the left side (negative valence or past time). If this association is particularly for optimistic participants, then the main effect of Associated Side should interact with the SOP2 scores such that the side effect is stronger for people with high scores.

## Materials and Methods

### Participants

The experiment was announced via a mass mailing of the University of Tübingen as an online experiment, programmed with the software JsPsych (Version 6.0; de Leeuw, 2015). A precondition for participating in this study was full age (i.e., 18 years or older) and German as mother tongue. To provide an incentive for participation, participants either received a course credit of 15 min or could take part in a raffle (3 coupons of €10 for the German Railway). 197 native speakers (mean age = 24.9 years, *SD* = 6.4 years) performed the experiment (155 females, 39 males, 3 diverse genders). The experiment consisted of two parts (see “Materials and Methods” section) and lasted about 5 min.

### Materials

The material of the experiment involved 32 experimental sentence pairs and 19 filler sentence pairs. The sentences in the experimental pairs were structured into an initial sentence fragment and a completion phrase and were available in two versions. In each version, the initial sentence fragments were identical for the two sentences in a pair but the completion phrases differed. In 16 of the 32 experimental pairs, the initial sentence fragment specified the deictic time of the described event (given dimension: time). Depending on the version, these fragments specified the described event to take part in the future (given value: future; associated side: right), or specified the described event to have taken part in the past (given value: past; associated side: left). For the remaining 16 experimental pairs, the initial sentence fragment involved a valence noun (given dimension: valence). Depending on the version, these fragments involved a noun with positive valence (given value: positive; associated side: right) whereas the other eight involved a noun with negative valence (given value: negative; associated side: left). The completion phrases of each sentence pair always differed in the dimension that was not given in the initial sentence fragment. For the sentence-pairs with the given dimension time, the completion phrases contained a valence noun, whereby one completion phrase contained a positive noun and the other a negative noun. For sentence-pairs with the given dimension valence, the completion phrases specified the deictic time of the described event, whereby one completion phrase specified the event to take part in the future and the other specified it to have taken part in the past. It should be noted that the two versions of each pair differing in the given values systematically differ with respect to the associated side (left vs. right), which is why we will use the term Side to refer to this manipulation in the “Design and Analyses” and “Results” sections. For a complete list of the experimental items together with translations into English, see the Supplementary Appendix.

Specifications concerning deictic time are straightforward and not subjective. The same, however, is not true for valence. When constructing the materials for the present study, we therefore took into consideration the valence ratings provided in the *Berlin Affective Word List Reloaded* (BAWL-R; Võ et al., 2009). In particular, in the initial sentence fragments for the pairs with the given dimension valence, we only included as valence words nouns that had received a mean rating value of below -1.5 or above +1.5 on a 7-point-Likert scale ranging from -3 (very negative) to +3 (very positive) in the BAWL-R. The same held for the valence words in the completion phrases of experimental sentence pairs with the given dimension time. In addition, we took care not to include words with a negative or positive valence in the initial sentence fragments of the pairs with given dimension time. We only included nouns that had received a mean rating value of 0 in the BAWL-R.

In addition to the 32 experimental sentence pairs, there were 19 filler pairs, also structured into an initial sentence fragment and a completion phrase. These were only available in one version. In contrast to the experimental sentence pairs, the two completions were not both sensible but rather one completion phrase comprised a sensical and one a non-sensical completion (e.g., *In the sky there are*…. *clouds/ants*).

To assess potentially relevant personality differences between our participants, we used the Optimism-Pessimism scale (SOP2; Kemper et al., 2012), which operates on two items with ratings on a 7-point-Likert scale ranging from 1 (not at all optimistic) to 7 (very optimistic), and 1 (not at all pessimistic) to 7 (very pessimistic), respectively. As suggested by Kemper, we averaged the responses to the two items after recoding the second item. This average gives the SOP2 score (1 = very pessimistic and 7 = very optimistic).

### Procedure

After giving informed consent, participants were presented with the two parts of the experiment. In the first part of the experiment, participants were presented with the initial sentence fragment of the sentence pairs and were asked to choose one of the two completion phrases of the respective pair. In the second part of the experiment, participants completed the two items of the SOP2.

More specifically, in each trial of the first part of the experiment, participants were presented with a fixation cross for 700 ms after which the initial sentence fragment appeared centered on the screen (black font on white background, size: 23). After participants had pressed the space bar, the two different completions of the respective pair appeared on the left vs. right part of the screen. Participants chose one of the completion phrases by pressing the f- or the j-key for the phrase on the left vs. right side, respectively. Which completion appeared on which side of the screen was randomized from trial to trial. For this task, participants were instructed to choose the completion phrase that seemed a better ending for the initial sentence fragment. After participants had pressed the f- or the j-key, the completion phrase disappeared and the next trial started.

Each participant saw 32 experimental pairs, 16 with the given dimension time (eight in each of the given value/associated side versions) and 16 with the given dimension valence (eight in each of the given value/associated side version). We created two lists that counterbalanced the given value for the experimental pairs across participants such that each participant only saw one version of each pair (either the one with the left or the right associated side). Each list also included 11 filler pairs whereby three were used as practice items in the beginning of the experiment (identical for the two lists) and 8 were presented intermixed with the experimental pairs (different for the two lists). The aim of presenting the filler items (that only had one sensical response) was to provide the possibility of detecting participants with random choice behavior. The order of the sentences was random for the participants. Also, which completion phrase was presented on which side of the screen was determined randomly for each trial.

### Design and Analyses

The factorial design of this study involved the two within-subject factors *Given Dimension* (time vs. valence) and *Associated Side* (left vs. right). For the sake of simplicity, we will call the two factors *Dimension* and *Side* in what follows. The dependent variable was the two-alternative choice corresponding to the completion phrase. Again, for the sake of simplicity, we will code the choices as left vs. right-side choices depending on whether the positive/future completion phrase (right-side choice) or the negative/past completion phrase (left-side choice) was selected. We conducted mixed-model analyses (Bates et al., 2015) and also computed *minF’* (see Equations 15 and 16 in Clark, 1973b) to evaluate the experimental results statistically. Because linear mixed-models sometimes do not converge, we cross-checked the results with the *minF’* analysis to reassure the statistical results obtained. All analyses were performed in R (R Core Team, 2017).

## Results

The filler items served as a performance check (e.g., whether participants were responding randomly). We only included participants who gave at least 75% correct responses to the filler items. According to this criterion, the data of 8 participants were excluded from the final data analysis. The performance level on these filler items of the remaining 189 participants was 96.50% (*SE* = 0.53%) correct. Overall, participants demonstrated a relatively balanced selection of left-side and right-side choices (46.05% left, 53.95% right).

**FIGURE 1:**
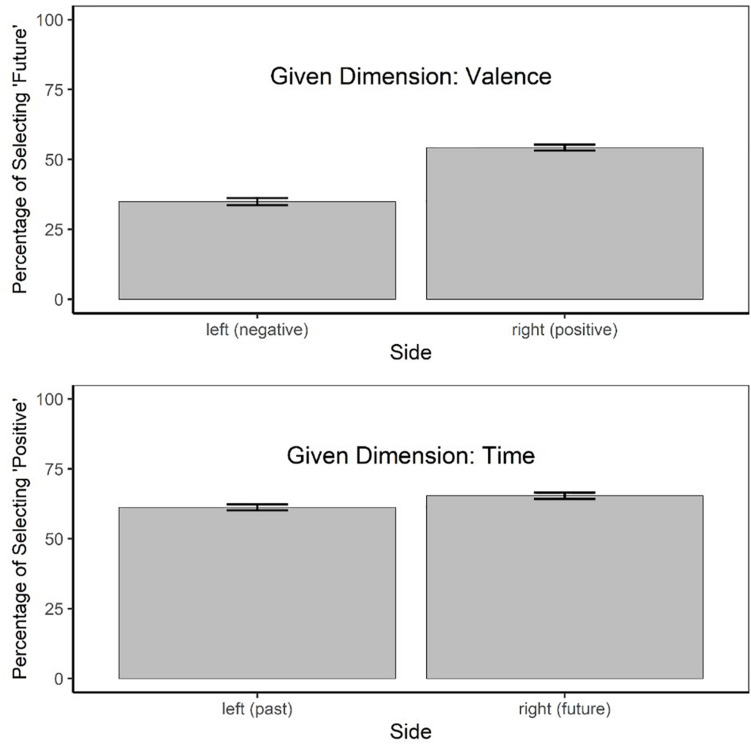
Upper Part: Percentage of selecting a future-related sentence completion phrase when the initial sentence fragment involved a negative or a positive valence word. Lower Part: Percentage of selecting a positive-related sentence completion when the initial sentence fragment related either to the past or to the future. The error bars depict 95% confidence intervals computed from the within-subject standard error of mean according to Cousineau (2007) with the correction suggested by Morey (2008).

Figure 1 depicts the major results of this study. The upper panel shows the percentage of selecting future completion phrases for the given dimension valence as a function of side (left: negative, right: positive). In contrast, the lower panel shows the percentage of chosen positive completion phrases for the given dimension time as a function of side (left: past, right: future).

There was a main effect of Dimension, *F*1(1, 188) = 223.62, *p* < 0.001, *F*2(1, 30) = 6.38, *p* = 0.017, *minF’*(1, 32) = 6.21, *p* = 0.018. The percentage of right-side choices was generally higher for the given dimension of time than for the given dimension of valence. In other words, participants selected more “positive” than “future” completions (63.29 vs. 44.61%). Most important, however, there was a reliable main effect of Side, *F*1(1, 188) = 102.23, *p* < 0.001, *F*2(1, 32) = 17.85, *p* < 0.001, *minF’*(1, 41) = 15.20, *p* < 0.001. As expected, choices associated with the right side (59.82%) were more frequent than those associated with the left side (48.08%). Moreover, there was also a significant interaction between the two factors, *F*1(1, 188) = 49.03, *p* < 0.001, *F*2(1, 30) = 7.60, *p* = 0.010, *minF’*(1, 40) = 6.58, *p* = 0.014. This interaction reflects a stronger effect of factor Side for dimension valence than for dimension time. Specifically, when the initial sentence fragment referred to the future, participants selected the positive alternative in 65.34%, whereas this percentage was 61.24% when the initial sentence referred to the past (paired Wilcoxon test, *V* = 6,846.5, *p* = 0.005). By contrast, when the initial sentence was positive, participants selected future-related sentence completions in 54.30% of the cases, whereas this percentage was 34.92% when the initial sentence fragment was negative (paired Wilcoxon test, *V* = 11,230, *p* < 0.001).

A mixed-model analysis yielded basically identical results as the above *minF’*-analysis. We used the function glmer of the “lme4” package (version 1.1-23) with the link function logit (i.e., family = binomial). To this end, we fitted various complex models until we obtained one that converged:^2^

DV∼1+SideDimension*+(1+Side+Dimension|Participant)+(1+Side|Item)

This restricted model version yielded a main effect of Dimension (β = 0.56, *SE* = 0.20, *z* = 2.76, *p* = 0.006), a main effect of Side (β = −0.31, *SE* = 0.07, *z* = −4.33, *p* < 0.001), and a significant interaction of the two factors (β = 0.16, *SE* = 0.07, *z* = 2.33, *p* = 0.020).

In a *post hoc* analysis, we included the SOP2 scores of the Optimism-Pessimism scale as a covariate to examine possible interindividual differences. The average SOP2 score was 4.62 (*SE* = 0.02). The scores correlated positively with the dependent variable (*r* = 0.19, *t* = 2.64, *df* = 187, *p* = 0.009), meaning that the selection of right-choices increased with increasing SOP2, that is, optimistic people tend to select future and positive completions over past and negative completions. Thus we also included SOP2 as a covariate in the above mixed-model,

DV∼1+Side⁢Dimension*⁢SOP2*+(1+Side+Dimension|Participant)+(1+Side|Item)

which significantly improved the model fit χ*^2^* = 20.24, *df* = 4, *p* < 0.001. This additional analysis not only revealed a main effect of this covariate (β = 0.10, *SE* = 0.03, *z* = 3.16, *p* < 0.001) echoing the above correlation but also an interaction with Dimension (β = 0.08, *SE* = 0.03, *z* = 2.86, *p* = 0.004) and with Side (β = 0.08, *SE* = 0.03, *z* = 3.02, *p* = 0.003). These interactions demonstrate that the main effects of Dimension and Side in the previous analyses are primarily driven by participants who score high on the Optimism-Pessimism scale.

**FIGURE 2:**
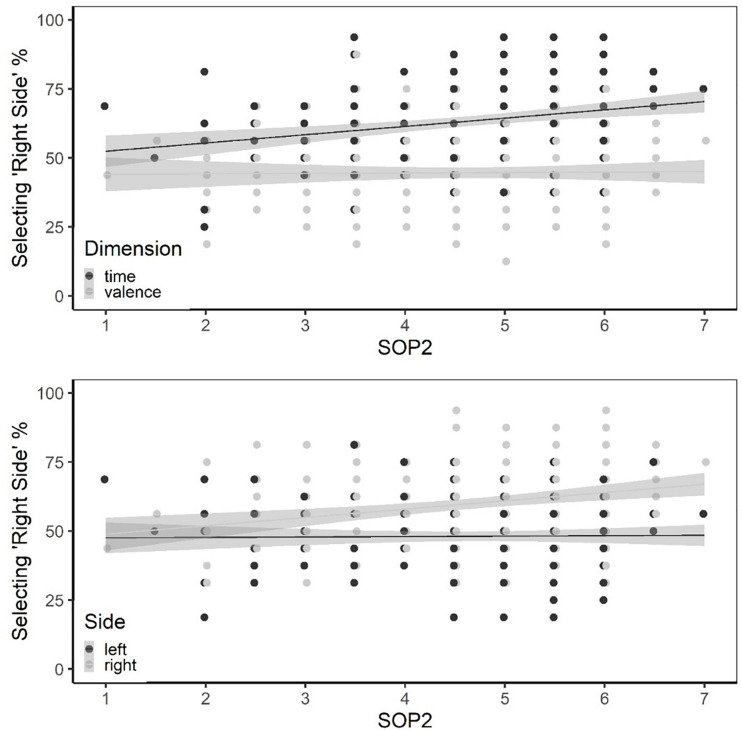
Upper panel: Illustration of interaction between SOP2 and Dimension. Lower panel: Illustration of interaction between SOP2 and Side. The lines represent best-fitting regression functions conditional on time or valence (upper panel) and conditional on the left- or right side (lower panel).

The upper panel in Figure 2 illustrates the interaction between SOP2 and Dimension. As the conditional regression functions in this panel reveal, the percentage of right-side choices increased gradually with SOP2 when the given dimension in the first segment was time but not when it was valence. The lower panel in Figure 2 exhibits the interaction between SOP2 and Side. When the first sentence fragment referred to the right side (i.e., positive valence or future), the percentage of right-side choices for the continuation fragment increased gradually with SOP2. However, as can also be seen in this panel, SOP2 did not modulate the frequency of right-side choices when the first sentence fragment referred to the left side.

## Discussion

In this study we investigated whether there is an association between deictic time and valence in the sense that future is linked to positive and past is linked to negative. We employed a new approach that does not involve explicit temporal or valence ratings and is, therefore, more implicit compared to previous approaches that studied this potential association. When participants read an initial sentence fragment that specified the deictic time of an event and were asked to choose a positive or negative completion phrase, then the percentage with which they chose a positive phrase was higher when the described event was to take place in the future compared to when it had taken part in the past. Similarly, when participants read an initial sentence fragment with a valence word and were asked to choose a completion phrase that locates the respective event in the past or the future, then the percentage with which they chose a future completion phrase was higher when the initial sentence fragment was positive compared to when it was negative. Both results fit well with the hypothesis that there is an association between time and valence, which links future time to positive valence and vice versa.

One remarkable aspect of our results was that the difference in the choices was larger when the given dimension was valence compared to when it was time. This asymmetry might be related to the fact that for sentences with time as given dimension, participants had to choose between a positive and a negative completion phrase. It seems well possible that there is a general tendency to select the positive over the negative, making any additional factors that influence the choice challenging to observe. In contrast, for sentences with the given dimension “valence,” participants had to choose between a future and a past completion phrase, and here it is not as evident that there should exist a general preference for one over the other.

Another remarkable aspect of our results is the strong modulation of the effects by the SOP2 values. It seems that the association between time and valence is pronounced for people with an optimistic attitude toward life. Interestingly, however, the association does not reverse for people with a more pessimistic attitude. Specifically, people with low SOP2 scores did not exhibit an association between time and valence, reflecting a link between future time and negative valence. For these people, the percentages with which they chose a right-side completion phrase did not differ between the given values in the initial sentence fragment. One possibility is that the association between time and valence is only partly determined by attitude (optimistic vs. pessimistic). Possibly, there is another more fundamental or cognitive basis for the association between time and valence that links future to positive and past to negative. If so, there might be two counteracting factors in individuals with a pessimistic attitude, one linking future to negative valence (pessimism) and one linking future to positive (cognitive), leading to the observed null difference for people with low SOP2 scores. In the next paragraph, we discuss one potential cognitive basis for the association between time and valence.

One way to explain the association between time and valence linking future to positive and past to negative suggests itself when considering metaphoric mapping accounts of abstract concepts. According to these accounts, abstract concepts such as time and valence are understood by mapping them onto a concrete dimension like space. According to the literature, time and valence are both mapped onto the lateral spatial axis. Future is linked to the right and past is linked to the left (Santiago et al., 2007; Ulrich and Maienborn, 2010). Positive valence is linked to the right and negative valence to the left, at least for right handers (Casasanto, 2009; de la Vega et al., 2012). Therefore, time and valence might be associated based on this common mapping onto the lateral spatial axis with future being linked to positive (via a common mapping onto the right) and past being linked to the negative (via a common mapping onto the left). Our results fit well with this explanation, especially when considering the possibility that this common spatial mapping affected the behavior in our sentence-continuation paradigm in addition to factors related to attitude differences. However, even if we assumed that there must be another basis for the observed association between time and valence besides individual differences, we cannot be sure that this is indeed rooted in a common spatial representation of time and valence. In principle, it also seems conceivable that there is a direct mapping between time and valence that might be genetically anchored. After all, linking future time and positive valence seems like a precondition for a motivated going on with one’s life and, therefore, might be a functional property of human beings. This possibility is consistent with those studies showing that imagined future events are more positive than past counterparts (e.g., Newby-Cark and Ross, 2003; D’Argembeau and Van der Linden, 2004; Berntsen and Bohn, 2010; Rasmussen and Berntsen, 2013).

How could one investigate experimentally whether a common spatial representation lies at the bottom of the association between time and valence? We think that investigations involving left-handers would be particularly informative for this issue. In contrast to right-handers, left-handers link positive valence to the left (their dominant hand) and negative valence to the right (their non-dominant hand), exhibiting the exact opposite mapping of valence to space compared to right-handers (Casasanto, 2009; de la Vega et al., 2012). Thus, if the mapping of time and valence has its roots in a common mapping of time and valence onto the lateral spatial axis, we should see different associations between time and valence for left- and right-handers. Right-handers should link future to positive and past to negative. For left-handers, this should either be the opposite (linking future to negative and past to positive) or the association observed for right-handers should be less pronounced. In our present study, we did not collect handedness information. However, it is likely that most of our participants were right-handed and this is consistent with the fact that we observed an association between time and valence linking future to positive and past to negative but not the other way around. Future studies are necessary to investigate in more detail the different bases of the observed time-valence association that takes into account personality differences as well as differences related to handedness.

## Conclusion

The two abstract concepts valence and time seem to be associated whereby future is linked to positive valence and past is linked to negative valence. This association is particularly pronounced for people with an optimistic personality. Future studies are needed to find out whether this association is also particularly pronounced for right- compared to left-handed people. If so, this would provide evidence that the association is at least partly rooted in a common mapping of time and valence onto the lateral spatial axis. Moreover, follow-up studies may also examine whether the present results generalize to cultures where deictic time runs from the right (past) to left (future) rather than from the left to the right like in Western cultures (Ouellet et al., 2010).

## Data Availability Statement

The datasets presented in this study can be found in online repositories. The names of the repository/repositories and accession number(s) can be found below: https://osf.io/qb8dp/.

## Ethics Statement

Ethical review and approval was not required for the study on human participants in accordance with the local legislation and institutional requirements. The patients/participants provided their written informed consent to participate in this study.

## Author Contributions

BK and NS designed the experiment. NS collected the data. NS, BK, and RU analyzed the data. BK and RU wrote the manuscript. All authors contributed to the article and approved the submitted version.

## Conflict of Interest

The authors declare that the research was conducted in the absence of any commercial or financial relationships that could be construed as a potential conflict of interest.

## Footnotes

1 https://www.merriam-webster.com/dictionary/optimist

2 For example, the model DV ∼ 1 + Side^∗^Dimension + (1 + Side^∗^Dimension | Participant) + (1 + Side | Item) did not converge.
