# Supplementary material for: Associations Between Abstract Concepts: Investigating the Relationship Between Deictic Time and Valence
Source: Front Psychol. 2021 Feb 12;12:612720. doi: 10.3389/fpsyg.2021.612720 (PMC7907156; doi:10.3389/fpsyg.2021.612720)
Supplement: Supplementary file 1 [file Table_1.pdf]

Supplementary Material for Kaup, Scherer, & Ulrich: Associations between abstract concepts: Investigating the relationship between deictic time and valence

Items with the given dimension Valence

No.	Given Value	Sentence Fragment	Completion: Past	Completion: Future
**1**	**Pos**	Die Übergabe des Geschenkes	ist vorüber	steht aus
	**Neg**	Die Übergabe des Revolvers	ist vorüber	steht aus
		The handover of the gift is over/ is pending. The handover of the revolver is over / is pending.		
**2**	**Pos**	Die Feier meines Kollegen	fand gestern statt	wird morgen stattfinden
	**Neg**	Der Abschied meines Kollegen	fand gestern statt	wird morgen stattfinden
		My colleague's party took place yesterday / will take place tomorrow. My colleague's farewell took place yesterday / will take place tomorrow.		
**3**	**Pos**	Das Vorspiel	ist vorbei	steht bevor
	**Neg**	Das Verbrechen	ist vorbei	steht bevor
		The foreplay is over / is imminent. The crime is over / is imminent		
**4**	**Pos**	Mein Mut	kam letzte Woche zum Vorschein	wird nächste Woche zum Vorschein kommen
	**Neg**	Meine Psychose	kam letzte Woche zum Vorschein	wird nächste Woche zum Vorschein kommen
		My courage came to light last week / will come to light next week. My psychosis came to light last week / will come to light next week.		
**5**	**Pos**	Eine Umarmung	bekam Franz gestern	wird Franz morgen bekommen
	**Neg**	Eine Ohrfeige	bekam Franz gestern	wird Franz morgen bekommen
		Franz got a hug yesterday / A hug will be given to Franz tomorrow. Franz got a slap in the face yesterday / Franz will get a slap in the face tomorrow.		
**6**	**Pos**	Die Führung durch den Palast	fand letzte Woche statt	wird nächste Woche stattfinden
	**Neg**	Die Führung durch den Bunker	fand letzte Woche statt	wird nächste Woche stattfinden
		The tour through the palace took place yesterday / will take place tomorrow. The tour through the bunker took place yesterday / will take place tomorrow.		
**7**	**Pos**	Die Party	war gestern	steht bevor
	**Neg**	Der Streit	war gestern	steht bevor
		The party was yesterday / is imminent. The dispute was yesterday / Is imminent.		
**8**	**Pos**	Die Ankunft an der Küste	war gestern	steht bevor
	**Neg**	Die Ankunft bei der Sekte	war gestern	seht bevor
		The arrival on the coast was yesterday / is imminent. The arrival at the sect was yesterday / is imminent.		
**9**	**Pos**	Die Erholung	erfolgte letzten Monat	wird nächsten Monat erfolgen
	**Neg**	Der Verlust	erfolgte letzten Monat	wird nächsten Monat erfolgen
		The recovery took place last month / will take place next month. The loss occurred last month / will take place next month.		

**10**	**Pos**	Das Konzert	fand gestern statt	findet morgen statt
	**Neg**	Die Spionage	fand gestern statt	findet morgen statt
		The concert took place yesterday / will take place tomorrow. The espionage took place yesterday / will take place tomorrow.		
**11**	**Pos**	Die Ferien	waren letzte Woche	sind nächste Woche
	**Neg**	Die Attentate	waren letzte Woche	sind nächste Woche
		The vacation as last week / is next week. The assassinations were last week / are next week.		
**12**	**Pos**	Das Treffen mit der Geliebten	war gestern	ist für morgen geplant
	**Neg**	Das Treffen mit dem Zahnarzt	war gestern	ist für morgen geplant
		The meeting with the beloved was yesterday / is planned for tomorrow. The meeting with the dentist was yesterday / is planned for tomorrow.		
**13**	**Pos**	Die Erdbeeren	wurden gestern gesammelt	werden morgen gesammelt
	**Neg**	Die Granaten	wurden gestern gesammelt	werden morgen gesammelt
		The strawberries were collected yesterday / will be collected tomorrow. The grenades were collected yesterday / will be collected tomorrow.		
**14**	**Pos**	Das Lob	wurde gestern ausgesprochen	wird morgen ausgesprochen
	**Neg**	Die Lüge	wurde gestern ausgesprochen	wird morgen ausgesprochen
		The praise was expressed yesterday / will be expressed tomorrow. The lie was expressed yesterday / will be expressed tomorrow.		
**15**	**Pos**	Die Extase	überkam mich gestern	wird mich morgen überkommen
	**Neg**	Die Übelkeit	überkam mich gestern	wird mich morgen überkommen
		Ecstasy came over me yesterday / will come over me tomorrow. The nausea came over me yesterday / will come over me tomorrow.		
**16**	**Pos**	Annas Zusage	wurde gestern verkündet	wird morgen verkündet
	**Neg**	Annas Zweifel	wurde gestern verkündet	wird morgen verkündet
		Anna's promise was announced yesterday / will be announced tomorrow. Anna's doubts was announced yesterday / will be announced tomorrow.		

Items with the given dimension Time

No.	Given Value	Sentence Fragment	Completion: Negative	Completion: Positive
**17**	**Future**	Bei der Verlosung letzte Woche	machte Franz Verlust	machte Franz Gewinn
	**Past**	Bei der Verlosung nächste Woche	wird Franz Verlust machen	wird Franz Gewinn machen.
		At last week's raffle Franz made a loss / made a win. At next week's raffle Franz will make a loss / will make a win.		
**18**	**Future**	Gestern las Gabi über das Thema	Faschismus	Sex
	**Past**	Morgen wird Gabi über das Thema	Faschismus lesen	Sex lesen
		Yesterday, Gabi read about fascism / about sex. Tomorrow, Gabi will read about fascism / about sex.		
**19**	**Future**	Der Vortrag letzte Woche behandelte das Thema	Abgas	Licht
	**Past**	Der Vortrag nächste Woche wird das Thema	Abgas behandeln	Licht behandeln
		Last week's lecture dealt with the topic of exhaust gas / light. Next week's lecture will deal with the topic of exhaust gas / light.		
**20**	**Future**	Letzen Monat war die Arbeit geprägt von	Konflikten	Einigungen
	**Past**	Nächsten Monat wird die Arbeit geprägt sein von	Konflikten	Einigungen
		Last month, the work was characterized by conflicts / agreements. Next month, the work will be characterized by conflicts / agreements.		
**21**	**Future**	Die Predigt letzte Woche handelte von	Armut	Erfüllung
	**Past**	Die Predigt nächste Woche wird von	Armut handeln	Erfüllung handeln
		The sermon last week was about poverty / fulfilment. The sermon next week will about poverty / fulfilment.		
**22**	**Future**	Letze Woche machten wir mit der Gruppe	Ärger	Waffeln
	**Past**	Nächste Woche werden wir mit der Gruppe	Ärger machen	Waffeln machen
		Last week we caused trouble / made waffles with the group. Next week we will cause trouble / make waffles with the group.		
**23**	**Future**	Letztes Jahr im Baltikum besuchten wir	das Ghetto	den Palast
	**Past**	Nächstes Jahr im Baltikum werden wir	das Ghetto besuchen	den Palast besuchen
		Last year in the Baltic states, we visited the ghetto / palace. Next year in the Baltic states, we will visit the ghetto / palace.		
**24**	**Future**	Das Horoskop sagte letzten Monat	einen Verlust voraus	eine Chance voraus
	**Past**	Das Horoskop sagt nächsten Monat	einen Verlust voraus	eine Chance voraus
		The horoscope predicted a loss / an opportunity last month. The horoscope will predict a loss / an opportunity next month		
**25**	**Future**	Im Theater spielte Alex letztes Jahr	einen Schurken	ein Genie
	**Past**	Im Theater spielt Alex nächstes Jahr	einen Schurken	ein Genie
		Alex played a villain / genius in the theatre last year. Alex will play a villain / genius in the theatre next year.		

**26**	**Future**	Bei dem gestrigen Treffen auf dem Mini-Golf-Platz	lag Abfall	lag Schnee
	**Past**	Bei dem morgigen Treffen auf dem Mini-Golf-Platz	lag Abfall	lag Schnee
		At yesterday's meeting at the miniature golf course, there was garbage / snow. At tomorrow's meeting at the …, there will be garbage / snow		
**27**	**Future**	Gestern beim Nachbar sprachen wir über seine	Übelkeit	Gesundheit
	**Past**	Morgen werden wir beim Nachbar über seine	Übelkeit sprechen	Gesundheit sprechen
		Yesterday at the neighbour, we talked about his nausea / health. Tomorrow at the neighbour, we will talk about his nausea / health.		
**28**	**Future**	An dem Tisch roch es gestern nach	Gestank	Blumen
	**Past**	An dem Tisch wird es morgen nach	Gestank riechen	Blumen riechen
		At the table yesterday, it smelled bad / it smelled like flowers. At the table tomorrow, it will smell bad / will smell like flowers.		
**29**	**Future**	Beim Erledigen von Dingen hatte ich letzte Woche	Zeitnot	Ruhe
	**Past**	Beim Erledigen von Dingen werde ich nächste Woche	Zeitnot haben	Ruhe haben
		Last week, I had a shortage of time / enough time when doing my chores. Next week, I will have a shortage of time / enough time when doing my chores.		
**30**	**Future**	Das Gespräch mit meinem Chef letzten Mittwoch ging um	ein Problem	einen Wunsch
	**Past**	Das Gespräch mit meinem Chef nächsten Mittwoch wird um	ein Problem gehen	einen Wunsch gehen
		The conversation with my boss last Wednesday was about a problem / wish. The conversation with my boss next Wednesday will be about a problem / wish.		
**31**	**Future**	Letze Woche ging ich zu	dem Zahnarzt	meiner Tochter
	**Past**	Nächste Woche gehe ich zu	dem Zahnarzt	meiner Tochter
		Last week I went to the dentist / my daughter. Next week I will go to the dentist / my daughter.		
**32**	**Future**	Wir sahen uns gestern im	Stau	Garten
	**Past**	Wir werden uns morgen im	Stau sehen	Garten sehen
		We saw each other in a traffic jam / in the garden yesterday. We will see each other in a traffic jam / the garden tomorrow.		
